# A global map of dominant malaria vectors

**DOI:** 10.1186/1756-3305-5-69

**Published:** 2012-04-04

**Authors:** Marianne E Sinka, Michael J Bangs, Sylvie Manguin, Yasmin Rubio-Palis, Theeraphap Chareonviriyaphap, Maureen Coetzee, Charles M Mbogo, Janet Hemingway, Anand P Patil, William H Temperley, Peter W Gething, Caroline W Kabaria, Thomas R Burkot, Ralph E Harbach, Simon I Hay

**Affiliations:** 1 Spatial Ecology and Epidemiology Group, Tinbergen Building, Department of Zoology, University of Oxford, South Parks Road, Oxford OX1 3PS, UK; 2 Public Health and Malaria Control Department, PT Freeport Indonesia, Kuala Kencana, Papua, Indonesia; 3 Institut de Recherche pour le Développement, Lab. d'Immuno-Physiopathologie Moléculaire Comparée, UMR-MD3/Univ. Montpellier 1, Faculté de Pharmacie, 15, Ave Charles Flahault, 34093 Montpellier, France; 4 BIOMED, Universidad de Carabobo, Apartado 2073, Maracay 2101-A, Venezuela; 5 Laboratorio de Ecología de Vectores, Dirección de Control de Vectores y Fauna Nociva, Ministerio del Poder Popular para la Salud, Maracay, Venezuela; 6 Department of Entomology, Faculty of Agriculture, Kasetsart University, Bangkok, Thailand; 7 Malaria Entomology Research Unit, School of Pathology, Faculty of Health Sciences, University of the Witwatersrand, Johannesburg, South Africa; 8 Vector Control Reference Unit, National Institute for Communicable Diseases of the National Health Laboratory Service, Private Bag X4, Sandringham 2131 Johannesburg, South Africa; 9 KEMRI/Wellcome Trust Programme, Centre for Geographic Medicine Research - Coast, Kilifi, Kenya; 10 Liverpool School of Tropical Medicine, Liverpool, UK; 11 Malaria Public Health and Epidemiology Group, Centre for Geographic Medicine, KEMRI - Univ. Oxford - Wellcome Trust Collaborative Programme, Kenyatta National Hospital Grounds, P.O. Box 43640-00100, Nairobi, Kenya; 12 School of Public Health, Tropical Medicine and Rehabilitation Sciences, Queensland, Tropical Health Alliance, James Cook University, Queenland, Australia; 13 Department of Entomology, Natural History Museum, Cromwell Road, London SW7 5BD, UK; 14 Fogarty International Center, National Institutes of Health, Bethesda MD 20892, USA

## Abstract

**Background:**

Global maps, in particular those based on vector distributions, have long been used to help visualise the global extent of malaria. Few, however, have been created with the support of a comprehensive and extensive evidence-based approach.

**Methods:**

Here we describe the generation of a global map of the dominant vector species (DVS) of malaria that makes use of predicted distribution maps for individual species or species complexes.

**Results:**

Our global map highlights the spatial variability in the complexity of the vector situation. In Africa, *An. gambiae, An. arabiensis *and *An. funestus *are co-dominant across much of the continent, whereas in the Asian-Pacific region there is a highly complex situation with multi-species coexistence and variable species dominance.

**Conclusions:**

The competence of the mapping methodology to accurately portray DVS distributions is discussed. The comprehensive and contemporary database of species-specific spatial occurrence (currently available on request) will be made directly available via the Malaria Atlas Project (MAP) website from early 2012.

## Background

Global malaria vector maps, by necessity, must simplify a complex diversity of numerous interacting and sympatric anopheline species. Such simplification refines the information down to a minimum, indicating only the primary vector(s) at each location and provides users, such as public health officials, modellers and opinion formers, with a global and regional picture that is easy to digest and utilise for scientific, operational and advocacy purposes.

Global maps have long been used to aid in visualising the malaria problem. These include the vector species map of May [1] and the 12 zones of malaria epidemiology described by Macdonald [2], determined using broad climatic ranges and physical land features, as well as consideration of the known distribution of the major anopheline vectors at the time. More recently Mouchet *et al*. [3] updated Macdonald's map, reassigning the 12 zones into more conventional biogeographical regions. This history of malaria vector (or vector-associated) visualisation indicates a past appetite for such maps, continuing more recently with Kiszewski *et al*. [4] publishing a global distribution map for the major malaria vectors in 2004. Their map was created to aid the authors in the development of a malaria transmission 'stability' map, but has since been adopted widely within the malaria research community and reproduced in many publications (their paper is listed as being cited 81 times in Web of Science and 37 times in PubMED). There is, therefore, a substantial and continuing demand for global maps of the major vectors of malaria.

Human malarial protozoa are transmitted by mosquitoes of the genus *Anopheles*, which includes 465 formally recognised species and more than 50 unnamed members of species complexes [5]. Approximately 70 of these species have the capacity to transmit human malaria parasites [6] and 41 are considered here to be dominant vector species/species complexes (DVS), capable of transmitting malaria at a level of major concern to public health [7].

A comprehensive database of contemporary occurrence data for the 41 DVS was compiled over two years, beginning in January 2008 [7-10]. Using these and other data (see methods), distribution maps were produced for each species or species complex, which have been made available for download via the Malaria Atlas Project (MAP) website [11]. This paper describes the production of a global map of dominant malaria vectors using these individual species maps. No other published vector map has had the benefit of the extensive and comprehensive evidence base that underlies the work presented here.

## Methods

A full description of the species selection, data collection, database, modelling methodology and individual species map development is given elsewhere [7-10].

### Foundation maps

A list of 41 DVS were identified by consulting a number of authoritative reviews [3,4,12-14] ([3] now updated and translated [15]), including that of Kiszewski *et al*. [4], and included all those species or species complexes that were identified as 'principal', 'main', or 'dominant' vectors of malaria. Occurrence data for these 41 DVS were assembled into the MAP [7] vector database, incorporating published records of contemporary (post 1985) species-specific location information (Table 1). The database includes over 4800 sources relating to 15837 occurrence data points and also holds the other elements required to produce predictive distribution maps for all species/species complexes including a suite of open access environmental or climatic variables and expert opinion (EO) range maps.

**Table 1:** The 41 dominant vector species/species complexes (DVS) per region

Anopheline species or species complex	presence points
***Americas***	
*An. freeborni *Aitken	37
*An. pseudopunctipennis *Theobald*	156
*An. quadrimaculatus *Say*	379
*An. albimanus *Wiedemann	362
*An. albitarsis *Lynch Arribálzaga*	138
*An. aquasalis *Curry	57
*An. darlingi *Root	318
*An. marajoara *Galvão & Damasceno	56
*An. nuneztovari *Gabaldon*	171
**Total DVS: 9**	**1674**

***Europe & Middle-East***	
*An. atroparvus *van Thiel	1044
*An. labranchiae *Falleroni	234
*An. messeae *Falleroni	903
*An. sacharovi *Favre	183
*An. sergentii *(Theobald)	35
*An. superpictus *Grassi	385
**Total DVS: 6**	**2784**

***Africa***	
*An. arabiensis *Patton	1196
*An. funestus *Giles	919
*An. gambiae *Giles	1443
*An. melas *Theobald^‡^	149
*An. merus *Dönitz^‡^	73
*An. moucheti *Evans^‡^	66
*An. nili *(Theobald)*^‡^	105
**Total DVS: 7**	**3951**

***Asia***	
*An. barbirostris *van der Wulp*	872
*An. lesteri *Baisas & Hu	47
*An. sinensis *Wiedemann	568
*An. aconitus *Dönitz^† ‡^	424
*An. annularis *van der Wulp^† ‡^	496
*An. balabacensis *Baisas	14
*An. culicifacies *Giles*	550
*An. dirus *Peyton & Harrison*	372
*An. farauti *Laveran*	1465
*An. flavirostris *(Ludlow)	103
*An. fluviatilis *James*	83
*An. koliensis *Owen	325
*An. leucosphyrus *Dönitz/*latens *Sallum & Peyton	12
*An. maculatus *group	471
*An. minimus *Theobald*	445
*An. punctulatus *Dönitz*	379
*An. stephensi *Liston	261
*An. subpictus *Grassi*^† ‡^	410
*An. sundaicus *(Rodenwaldt)*	131
**Total DVS: 19**	**7428**

**TOTAL: 41**	**15837**

*Species complex; ^†^Not included in multi-species maps; ^‡^Not included in global map

Using the boosted regression tree (BRT) modelling methodology [16], predicted distribution maps were produced for each DVS. Nine species/species complex maps were produced for the Americas, seven for Africa, six for Europe and the Middle East and 19 for the Asian-Pacific region.

### Building the global map

In ArcMap [17], each predictive species map was buffered at a 50 km limit beyond the EO boundary and only those pixels with a presence probability greater than 0.5 were included.

On a country-by-country basis, and by region, a list of all the DVS known and predicted to occur in each malaria endemic country was created. The lists were circulated to the project Technical Advisory Group (TAG) who identified the three most important DVS per country (where there were three or more species) and ranked these species by their relative importance. Due to the known complexity of DVS found in the Asian-Pacific region, all DVS found in each country within the region were ranked by importance. Additionally, for some countries (e.g. Indonesia), where the importance of a vector species can vary greatly across a country's geographical extent, more detailed species-specific spatial information was gathered. These rankings were used to guide the creation of the multi-species maps where every attempt was made to ensure that the top ranked species in specific countries and regions were uppermost in the map species layers (i.e. were displayed preferentially over the less highly ranked species). In those areas where the dominance of one species is not clear (e.g. *An. arabiensis, An. funestus, An. gambiae *across sub-Saharan Africa), the predictive maps for each were merged to indicate the presence of a combination of equally dominant species.

In the Asian-Pacific region, *An. subpictus s.l., An. aconitus *and *An. annularis*, which were all included in the original list of 41 DVS (Table 1), are not shown on the multi-species maps. *Anopheles aconitus *and *An. annularis *both tend to play only a focal role in malaria transmission within their respective ranges and are often considered secondary or incidental [18-20], however, in 'ideal conditions' *An. aconitus *can be a major DVS and similarly, *An. annularis *is important only in selected areas in India, Sri Lanka and Nepal. Both species are essentially zoophilic [19,21,22], as is *An. subpictus s.l*. and with this latter DVS, there is question about the reported identification of specimens based only on morphological characteristics. Thus in the assessment of dominance in this region, these three species were ranked lowest and therefore when represented on the multi-species map, were overlaid completely by other more important species and were removed.

Due to the fine detail available for each of the predictive maps, in addition to the global distribution, maps are also presented for those regions that contain countries with a high burden of malaria, i.e. the Americas, Africa and the Asian-Pacific region. Moreover, additional maps are also provided which highlight areas where there is a particularly high diversity of vector species (e.g. Central America, South-East Asia and Pacific).

Finally, two maps are presented for the African region. The first illustrates the distribution of those species considered to be of major importance, even within the confines of the DVS ranking (i.e. *An. gambiae, An. arabiensis, An. funestus*). Each of these species has a large range that would obscure the other DVS present in Africa. Moreover, as these species tend to be the focus of most vector control efforts, it seemed prudent to provide a map that only indicates their distributions. However, this does not mean that the other DVS in Africa should be overlooked. For example, in the forested areas of western/central Africa *An. moucheti *is known to be a highly anthropophilic and efficient vector. Therefore a second map of Africa dedicated to showing the distributions of these 'secondary' DVS (in comparison to the 'top three') is also presented.

## Results

**Figure 1:**
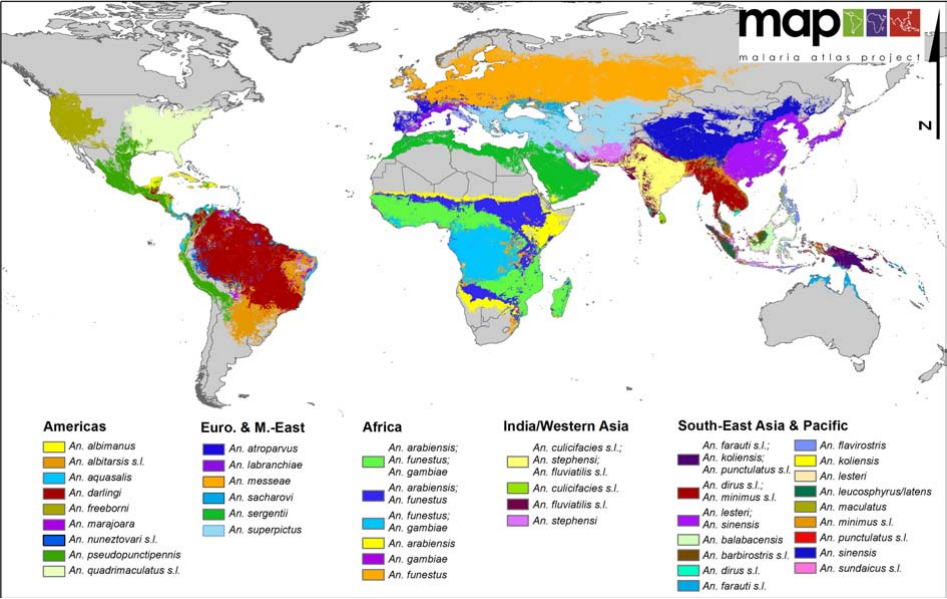
**A global map of dominant malaria vector species**.

**Figure 2:**
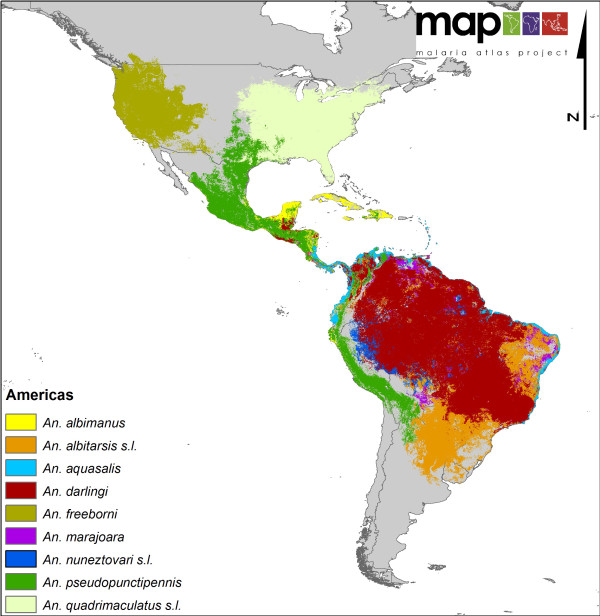
**A regional map showing the distribution of nine DVS across the Americas**.

**Figure 3:**
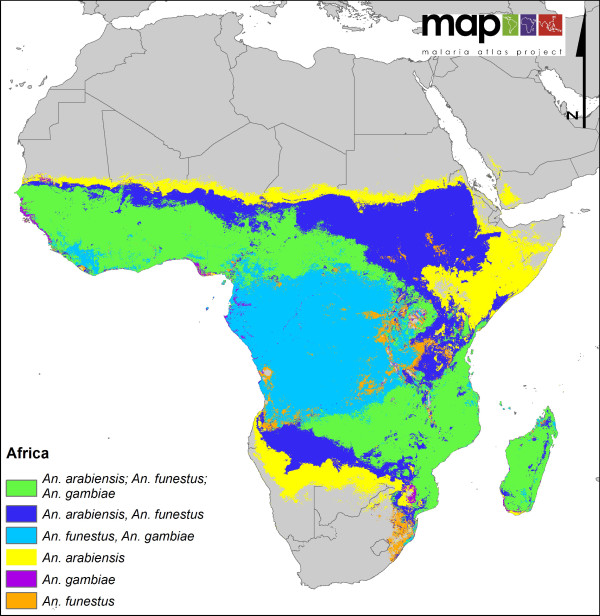
**A regional map showing the distribution of the three most dominant malaria vectors in Africa**.

**Figure 4:**
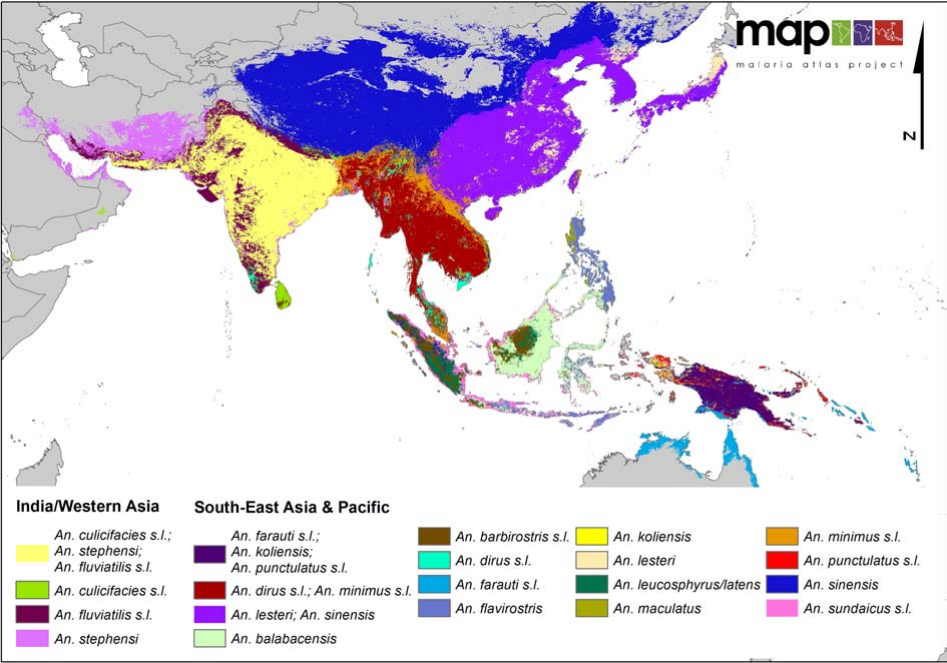
**A regional map showing the distribution of 16 dominant malaria vectors in the Asian-Pacific region**.

**Figure 5:**
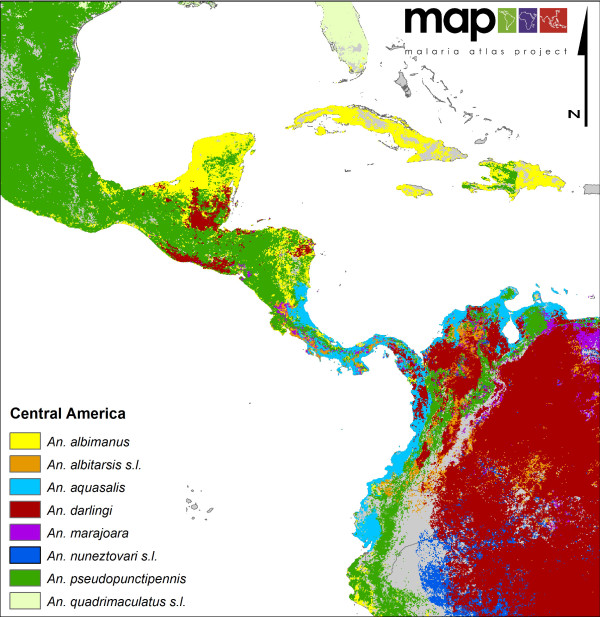
**A map showing a closer view of the complexity and diversity of the distribution of eight DVS in Central America and in the northern regions of South America**.

**Figure 6:**
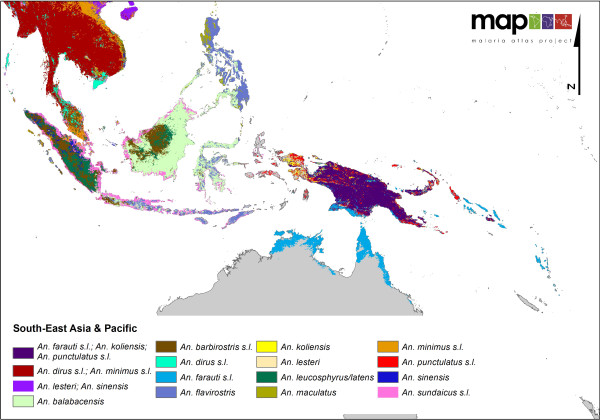
**A map showing a closer view of the complexity and diversity of the DVS in Southeast Asia and on the Pacific islands**.

**Figure 7:**
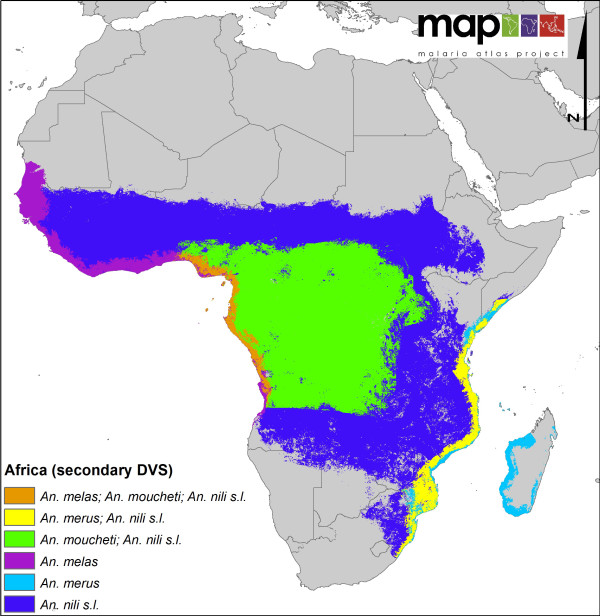
**A map showing the distribution of 'secondary' DVS across Africa**.

The number of presence data per species or species complex within the database included in the original model is given in Table 1. The global map indicating the distribution of 34 DVS is presented in Figure 1 (Figure 1 is also provided as a downloadable poster in Additional file 1). More detail is provided in the regional maps (Figures 2, 3, 4), which present a larger scale image of the information given in the global map. Figures 5 &6 provide maps focused specifically on areas of high DVS diversity including Central America (Figure 5) and South-East Asia and Pacific Islands (Figure 6). Figure 7 indicates the secondary DVS found in Africa that were not included on the global map (Figure 1).

Figure 1 highlights the variability in the complexity of the malaria vector species communities and their distribution on a global scale. For example, comparing the Asian-Pacific region with Africa clearly demonstrates the highly complex and diverse nature of the DVS in Asia, whereas Africa shows a relatively simple picture, with the three main species co-dominating western (except the forested west of the Democratic Republic of Congo (DRC), Republic of Congo, Angola etc. where *An. arabiensis *is not found) and southeastern areas. The ability of *An. arabiensis *to utilise drier environments than *An. gambiae *or *An. funestus *is also clearly indicated, with the distribution of *An. arabiensis *extending farther north into the Sahel, east into Ethiopia, the southwestern corner of the Arabian Peninsula, Kenya and Somalia and south into the desert and steppe environments of Namibia and Botswana in southern Africa.

Across Europe and the Middle East, the most striking feature is the extent of the range of *An. messeae *that extends from the United Kingdom in the west across western and eastern Europe into Asia. This species is also the most northerly distributed of the 34 DVS.

North America (Figure 2) shows a very simple vector profile, with only *An. freeborni *found in the northwest and *An. quadrimaculatus s.l*. in the southeast with some minimal overlap with *An. pseudopunctipennis *in the very south of the continent. In South America, *An. darlingi *is shown as mainly dominant, but in Central America (where *An. darlingi *is also present but more focally distributed) its dominance is superseded by *An. albimanus *and *An. pseudopunctipennis. Anopheles aquasalis *is not a particularly efficient vector, but its ability to oviposit and utilise saline larval habitats means it remains the 'dominant' species in coastal areas of Central and South America.

An additional and important contribution of the current DVS map in South America is that it highlights the presence of *An. marajoara*, an important emerging vector in both the Guyana shield and the Amazon basin, as well as the distribution of the often overlooked *An. albitarsis *complex in the savanna ecoregion [23].

In Asia (Figure 4 &6), the co-dominance of species is even more pronounced than found on the African continent, with the *An. culicifacies *complex, *An. stephensi *and the *An. fluviatilis *complex sympatric in India. The *An. dirus *and *An. minimus *complexes dominate together across much of Southeast Asia. However, along the Thai/Malaysian peninsula where these species both occur, they appear to have diverged and no longer occupy the same locations or ecological niche.

The Pacific islands (Figure 6) show a highly complex vector situation. On the island of New Guinea, members of the Punctulatus Group dominate, including the *An. farauti *complex, *An. koliensis *and the *An. punctulatus *complex, but only the *An. farauti *complex extends eastward to the Solomon Islands. Members of this complex are also found on the northern coast of Australia in Queensland and the Northern Territory.

In Indonesia, there appears to be high diversity and sympatry of vector species on the major islands. For example, in Sumatra, *An. sinensis *is found inland along with the *An. barbirostris *complex, *An. leucosphyrus/An. latens *and the *An. minimus *complex. A number of other species also exist on Sumatra, for example, the *An. maculatus *group and *An. flavirostris*, but none are considered dominant on the island; hence they are overlaid by the other, more dominant species. Alongside the *An. sundaicus *complex distributed along the coast, *An. flavirostris *does increase in relative 'dominance', by virtue of a reduced presence of other species, extending southward through Java until it is the only DVS found in the Lesser Sunda islands. In Sumatra, there is very little overlap amongst the dominant species found, suggesting that each occupies a separate niche on the island. *Anopheles balabacensis *dominates across most of Borneo, with some impact by the *An. barbirostris *complex and *An. leucosphyrus/latens *inland and the *An. sundaicus *complex on the coast.

In more northern areas of Asia (Figure 4), including China and Mongolia, *An. sinensis *and *An. lesteri *(syn. *An. anthropophagus*) are the only DVS. They appear to be sympatric in much of China and Korea, but this may be an artefact due to mis-identification of the two species in some areas.

## Discussion

### The data

The maps presented here show overlaid areas of species-specific predicted occurrence based on the climatic and environmental variables provided to the BRT model. Each species map included in the composite maps only included those pixels where the model predicted a probability of presence greater than 0.5. As with all species mapping, the quality of the output depends, for the most part, on the amount and quality of the data input into the model. Species occurrence data are often poorly distributed spatially [7-10] or are limited numerically (e.g. *An. leucosphyrus/An. latens *n = 12 (Table 1)). The modelling methodology allowed these data to be supplemented with randomly assigned (and therefore more spatially dispersed) pseudo-presence points taken from within the EO area of the species' range [10]. These pseudo-data were weighted at half that of the 'true' occurrence data. However, where the occurrence data were limited, the pseudo-data may have exerted a greater influence on the final model, and therefore on the area of predicted presence. This can be seen in the predicted species occurrence on New Guinea Island. The EO ranges for *An. farauti s.l., An. punctulatus s.l*. and *An. koliensis*, indicate a blanket coverage across the whole island without considering the highland areas that run across almost the entire central length of the island. Members of the Punctulatus Group, which include *An. farauti s.l., An. punctulatus s.l*. and *An. koliensis*, are not known to occur at altitudes higher than 2300 m (Bangs, unpub obs) and the highlands on this island peak with Puncak Jaya (Mt. Carstensz) at 4884 m [24]. The range of these three DVS centres on New Guinea island with limited spread to some of the other smaller neighbouring islands, and in the case of *An. farauti *s.l., to Northern Australia [8]. This small range may have focussed the pseudo-presence points which may have fallen within both the lower and higher altitude locations, and thus the model was unable to establish altitude as a limiting factor for these species.

The quality of the occurrence data also relies on accurate species identifications reported in the source literature. The data were faithfully abstracted from each source and no assumptions were made, however this will have introduced some varying level of error. For example '*An. funestus' *was rarely reported as a species complex, but also rarely subjected to the additional molecular methods of identification (e.g. Polymerase Chain Reaction (PCR)) [25,26] necessary to identify accurately the members of the complex. Moreover, it is possible that some studies were actually reporting more than one member of the Funestus Group or Subgroup rather than *An. funestus s.s*. or even the *An. funestus *complex. The same may also be said for the *An. maculatus *group in Asia.

For some species there is also current debate about their taxonomy; for example, the identity and vectorial capacity of *An. messeae *is currently in question, with some suggestion that *An. daciae *may be responsible for malaria transmission previously attributed to *An. messeae*, and may be sympatric with *An. messeae *across much of its range, which might also explain the apparent high polymorphism associated with *An. messeae *[27,28] .

Despite some uncertainty in species classifications that cannot be corrected, the presence points for each species were carefully examined by the TAG, and those points that were clearly unreliable or related to dubious species identifications were removed at an early stage in the mapping process.

### The maps

The maps presented here show the predicted occurrence of the DVS. They do not, however, indicate the probability of presence, although this information does underlie the distribution of positive and negative pixels (and is indicated on the original species maps [8-10]). A pixel is marked as 'present' where the BRT model indicated a probability of presence greater than 0.5. Therefore within these 'positives' the probability will range from > 0.5 to ≤ 1. Similarly, a pixel is marked as 'absent' where the BRT model indicated a probability of presence less than 0.5, but will include probabilities from 0 up to 0.5. These probability values are defined by the interaction of the environmental and climatic variables that are identified as predictors by the BRT model indicating where the environment is suitable for the species to exist. Hence such probabilities provide no direct information about potential species abundance but are simply the full output of the analysis. However, as these probabilities may indicate increasing or decreasing environmental suitability, it is feasible that these measures could be used to estimate species abundance at a specified location [29-31]. Further work is needed to try and establish a quantifiable link between these probabilities and DVS abundance.

Figure 1 provides the best currently available evidence-based global picture of the distributions of the main DVS. However, there will always be locations where the process has resulted in an oversimplification and the models do not pick up areas where a species may or may not be present. For example, in Africa, there is some question regarding the extensive predicted presence of *An. funestus *(species or members of the complex) within the highland areas of Ethiopia (Kiszewski, pers com). Indeed, elsewhere in the country, even where it is found, members of the Funestus Subgroup are rarely considered dominant, with *An. arabiensis *regarded as the major vector species [32]. Only one known study has conducted PCR identification of Funestus Group specimens from Ethiopia and only reported *An. parensis*, a non-vector, as present [33].

A lack of data across a large swath of central Africa should also be noted, for example only 3 sites reporting DVS occurrence were found for the Central African Republic, 2 sites in Congo and 23 in DRC [9]. Such areas therefore may not be being accurately represented by the model, especially where variable or unique environments and ecologies exist.

The large number of islands in the Asian-Pacific region, and those elsewhere of small size, can be problematic to accurately predict species occurrence. Overall, the models appear to have done well (based on TAG expert opinion), however there are a few cases where the model is not picking up areas of known presence. For example, on Grenada Island in the Americas the occurrence of *An. pseudopunctipennis *has been reported (see [10]) yet the model is not indicating a presence. However, *An. aquasalis *is correctly predicted to occur on this island. Similarly, *An. barbirostris s.l*. on the Lesser Sunda Island chain (including Flores, Sumbawa, Sumba, Timor and others) is not fully represented despite the existence of a published data point on Flores, and the islands being clearly within the EO range of this DVS. *Anopheles barbirostris s.l*. demonstrates dramatic varying behavioural attributes and vector importance over its geographical range in Indonesia, being of little or no epidemiological significance in Java and Sumatra in contrast to its role as a primary malaria vector in the eastern regions of the archipelago ([34,35] Bangs, unpub obs), thereby illustrating some of the difficulties with certain species and the finer details for interpreting distribution maps.

The scale of these regional and global maps can also limit the visibility of some areas of presence on the smaller islands. For example, the Maluku Island chain in eastern Indonesia, where *An. farauti s.l*. is an important vector along the coasts and *An. punctulatus s.l*. a vector inland, does indicate the presence of these vectors, but mostly as sporadic individual pixels, and thus their presence is easy to overlook.

The maps presented here show the predicted distributions of a number of species complexes without reference to the sibling species they represent. Moreover, the molecular forms (M and S) of *An. gambiae *are not distinguished despite reported behavioural differences between them. This is due to a lack of spatially dispersed data providing accurate and defendable sibling species or form identification. It is hoped that such data will become increasingly available as the importance of correctly and fully identifying these species becomes more widely accepted, thus allowing for updated and detailed species-specific maps to be produced in the future.

## Conclusions

Despite the known limitations and caveats given here, the global map represents the best currently available indication of the distributions of the dominant vectors of malaria. In line with the open access principles of the MAP, and via the ROADMAP initiative, the comprehensive dataset that was compiled to create the original species maps (currently available on request) will be directly available via the updated MAP website [11] from the beginning of 2012. The same site also hosts the individual species maps and bionomics information published in the earlier papers [8-10] and will also hold the maps published here in a format available for download.

### Future work

The global and regional maps presented here have been created with a range of users in mind including researchers, modellers, public health officials and vector control managers. The maps provide a good level of basic location information and highlight the most important vectors present in a particular area. However, for many of these stakeholders to be able to make use of this information fully, for example, in deciding the relevant control efforts needed at a particular location, they also need information on how these species and species complexes behave at that particular location.

An ongoing project conducted by MAP as part of the VECNet consortium [36] aims to address this need. Quantified and geo-located data on biology, propensity to infection, behaviour and larval site characteristics are being compiled, initially for *An. gambiae *but expanding to include other important DVS. These data will be made available via an online tool that will use both data from the maps presented here and data on species behaviour at different locations to facilitate control decision-making.

## Abbreviations

BRT: Boosted Regression Trees; DVS: Dominant Vector Species; EO: Expert Opinion; MAP: Malaria Atlas Project; PCR: Polymerase Chain Reaction; ROADMAP: Repository for Open Access Data collected by the Malaria Atlas Project; TAG: Technical Advisory Group.

## Competing interests

The authors declare that they have no competing interests.

## Authors' contributions

SIH conceived the study. MES compiled the global and regional multi-species maps, wrote the first draft of the manuscript and assembled the occurrence data, with assistance from CWK and others (see acknowledgements), and ran the models to create the individual DVS species maps. CWK also digitised and edited all the expert opinion maps. WHT designed and maintained the databases. APP implemented the BRT scripts for predictive mapping. PWG processed the environmental and climatic data grids. TAG members (MJB, SM, YR-P, REH, TC, MC, CMM and JH) provided data and advice in updating the EO range maps and in ranking the importance of the DVS per country. TRB provided additional comments on the maps. All authors participated in the writing and editing of the manuscript and read and approved the final submitted version. REH also served as the taxonomy advisor/consultant to the TAG and throughout this vector mapping project.

## Supplementary Material

Additional file 1 **Downloadable global and regional posters showing the distributions of DVS across the world**. Click here for file
