# Supplementary material for: A global map of dominant malaria vectors
Source: Parasit Vectors. 2012 Apr 4;5:69. doi: 10.1186/1756-3305-5-69 (PMC3349467; doi:10.1186/1756-3305-5-69)
Supplement: Additional file 1 — Downloadable global and regional posters showing the distributions of DVS across the world. [file 1756-3305-5-69-S1.PDF]

A global map of dominant malaria vector species

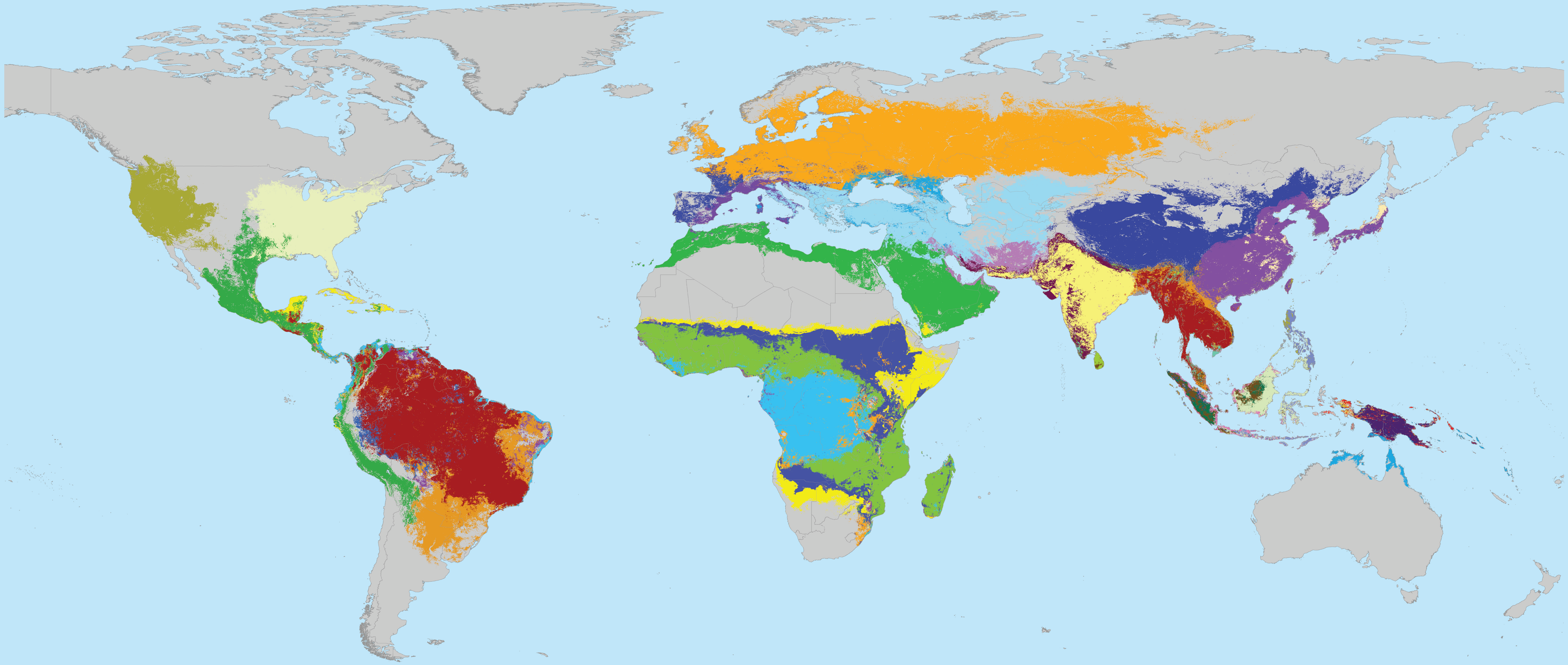

The Americas

- *An. darlingi*
- *An. aquasalis*
- *An. albitarsis s.l.*
- *An. marajoara*
- *An. nuneztovari s.l.*
- *An. pseudopunctipennis*
- *An. albimanus*
- *An. quadrimaculatus s.l.*
- *An. freeborni*

Euro. & M.East

- *An. superpictus*
- *An. sergentii*
- *An. sacharovi*
- *An. messeae*
- *An. labranchiae*
- *An. atroparvus*

Africa

- *An. arabiensis; An. funestus; An. gambiae*
- *An. arabiensis; An. funestus*
- *An. funestus; An. gambiae*
- *An. gambiae*
- *An. funestus*
- *An. arabiensis*

India/Western Asia

- *An. culicifacies s.l.; An. stephensi; An. fluviatilis s.l.*
- *An. fluviatilis s.l.*
- *An. stephensi*
- *An. culicifacies s.l.*

South-East Asia & Pacific

- *An. farauti s.l.; An. koliensis; An. punctulatus s.l.*
- *An. dirus s.l.; An. minimus s.l.*
- *An. lesteri; An. sinensis*
- *An. balabacensis*
- *An. barbirostris s.l.*
- *An. dirus s.l.*
- *An. farauti s.l.*
- *An. flavirostris*
- *An. koliensis*
- *An. lesteri*
- *An. leucosphyrus/latens*
- *An. maculatus*
- *An. minimus s.l.*
- *An. punctulatus s.l.*
- *An. sinensis*
- *An. sundaicus s.l.*

map malaria atlas project

VECNet

Parasites &Vectors

Sinka et al. *Parasites & Vectors*, 2012 5:69
